# Antibiotic exposure during weaning disrupts oral microbiota assembly in piglets

**DOI:** 10.1186/s40104-026-01483-y

**Published:** 2026-08-11

**Authors:** Cuipeng Zhu, Ping Hu, Peng Yuan, Jiajun Yu, Miaonan Zhu, Kennedy J. Ogamune, Hao Huang, In Ho Kim, Tlou G. Manyelo, Abdelkareem A. Ahmed, Demin Cai, Haoyu Liu

**Affiliations:** 1 Jiangsu Key Laboratory of Animal Genetic Breeding and Molecular Design, College of Animal Science and Technology, Yangzhou University, Yangzhou, 225009 People’s Republic of China; 2 Joint International Research Laboratory of Agricultural & Agri-Product Safety, The Ministry of Education of China, Yangzhou University, Yangzhou, People’s Republic of China; 3 Jiangsu Key Laboratory of Zoonosis, Yangzhou University, Yangzhou, People’s Republic of China; 4 Department of Animal Resource and Science, Dankook University, Cheonan, Republic of Korea; 5 Department of Agricultural Economics and Animal Production, University of Limpopo, Sovenga, South Africa; 6 Department of Veterinary Biomedical Sciences, Botswana University of Agriculture and Agriculture and Natural Resources, Gaborone, Botswana; 7 Guangling College of Yangzhou University, Yangzhou, 225100 People’s Republic of China

**Keywords:** Antibiotic exposure, IgA, Inflammation, Orotype, Oral microbiota, Weaning piglets

## Abstract

**Background:**

The use of antibiotics in swine production during the stressful weaning period is widespread. While their impact on the gut microbiome is documented, their effect on the developing oral microbiota, a critical gateway to systemic health, remains poorly understood. This study investigated how chronic exposure to tylosin (TYL) or a chlortetracycline-sulfadiazine-penicillin combination (CSP) shapes oral microbiota assembly in piglets from 21 to 60 days of age.

**Results:**

Healthy piglets exhibited a defined ecological succession, transitioning from an early, Pseudomonadota-dominated types of oral microbiota, or referred to as orotypes (driven by Moraxellaceae) at weaning to a stable, mature Bacillota-dominated state (driven by Lachnospiraceae) by 40 days of age. Antibiotic exposure disrupted this developmental program. CSP treatment locked the microbiota in an immature, Pseudomonadota-dominated state, while TYL promoted a dispersed and unstable Bacillota community. Dysbiosis was marked by enrichment of pathobionts (e.g., *Moraxella*, *Bergeyella*) and depletion of beneficial commensals like *Veillonella* and *Phocaeicola*, with the latter reduced in both the oral and gut microbiota. These structural shifts were linked to dysregulated microbial energy and lipid metabolism. Crucially, antibiotics compromised mucosal immunity, reducing salivary secretory IgA (SIgA), and provoked inflammation, evidenced by elevated salivary extracellular ATP (eATP), histological damage and transcriptome alteration in oral tissue, and changed serum metabolites.

**Conclusions:**

Our findings demonstrate that early-life antibiotic exposure disrupts the developmental programming of the oral ecosystem. The oral microbiota serves as a sensitive indicator of antibiotic impact and a key mediator of systemic health, highlighting the need for strategies that safeguard microbial succession to promote sustainable swine health.

**Supplementary Information:**

The online version contains supplementary material available at 10.1186/s40104-026-01483-y.

## Introduction

The global demand for high-quality animal protein is continuously increasing. Pork, in particular, is projected to reach a consumption level of 1.29 million tons in the coming years, constituting over one-third of total animal protein sources for humans [1]. To meet this demand, pig production has become highly industrialized. Its efficiency, therefore, depends largely on management practices, including the use of broad-spectrum antibiotics to mitigate bacterial infections, improve animal welfare, and enhance productivity [2–5]. This practice is especially prevalent during the weaning period, a critical phase for digestive tract development. At this stage, piglets face multiple stressors, including abrupt milk withdrawal, social reorganization, environmental changes, and the first ingestion of solid feed [2]. These events collectively predispose piglets to intestinal disorders, diarrhea and even mortality, leading to significant economic losses [6]. To combat weaning-associated diarrhea and infection, antibiotics are employed for their bactericidal or bacteriostatic effects, which target conserved cellular processes such as cell wall synthesis, membrane integrity, protein synthesis, and nucleic acid replication [7]. Although effective, early-life antibiotic exposure in piglets can cause collateral damage to commensal microbiota, resulting in long-term health consequences such as chronic disease and impaired immune function [8, 9]. Moreover, it poses substantial public health risks by promoting the dissemination of antimicrobial resistance (AMR) genes through selective pressure, contaminating the environment with resistant bacteria via horizontal gene transfer, and leaving drug residues in animal products [10, 11]. Alarmingly, AMR genes have been detected even in the oral microbiome of healthy individuals [12]. This is particularly relevant because antibiotics are most frequently administered via the oral route in animals. In humans, systemically absorbed antibiotics have been shown to be secreted in saliva and gingival crevicular fluid [13], exposing the oral microbiome to antimicrobials and underscoring their potential to disrupt its development, a key mediator of systemic health [14, 15].

Indeed, digestion begins in the mouth, which hosts the body's second-largest microbial community after the gut and comprises complex biofilms [16]. In mammals, this oral microbiota, a diverse consortium of bacteria, archaea, fungi, and viruses, plays a cardinal role in maintaining host homeostasis by functioning as a protective barrier, regulating metabolic exchanges, and interacting intricately with the host immune system [15]. Under physiological conditions, the porcine oral microbiome is primarily composed of Bacteroidota, Pseudomonadota, Bacillota, Actinomycetota, and Fusobacteriota, as described in piglets [17], and has been further resolved at the species level using metagenomic analysis yielding 202 metagenome-assembled genomes [18]. This equilibrium, however, is fragile and susceptible to perturbation [19]. Antibiotic administration is among the most potent disruptors of oral microbial community structure [20, 21]. In swine, various antibiotics alter gut microbiota composition; for example, tylosin transiently increases the abundance of Bacillota while accelerating its maturation [22], whereas a penicillin-chlortetracycline-sulfamethazine combination elevates Pseudomonadota and reduces Bacteroidota [9]. Critically, such effects extend beyond the gut. In mice, long-term systemic antibiotic exposure primarily disrupts the gut microbiota, which in turn drives oral dysbiosis via Th17-associated immune pathways, increasing the abundance of periodontal pathogens such as *Enterococcus* and *Dysgonomonas* [21]. Fecal microbiota transplantation can restore homeostasis in both niches, further illustrating the close linkage between gut and oral microbial communities. In pigs, this dysbiosis can shift the oral ecosystem from a commensal reservoir to a source of pathology. The oral cavity harbors potential pathogens like *Streptococcus suis*, which, although typically a commensal, can become invasive and cause septicemia in pigs [23], underscoring the vital importance of a stable oral microbial community for pig health and productivity. Alongside these direct microbial effects, antibiotics can further disrupt host homeostasis at multiple levels: by inducing metabolic alterations in the oral microbiota [24] and by impairing oral tolerance via gut-associated immune regulatory pathways. This vulnerability is particularly pronounced in young animals [25]. The oral cavity itself presents a critical and unique interface in this context. While it offers ideal conditions for microbial colonization, such as stable temperature, humidity, and abundant nutrients, it is also fortified by sophisticated host defense mechanisms [26]. These include the physical barrier of the acquired pellicle, the constant mechanical and antimicrobial cleansing action of saliva, and a specialized suite of immune mediators [27]. Foremost among these mediators are salivary immunoglobulins, particularly secretory IgA (SIgA) [28], and antimicrobial peptides, which collectively shape and constrain the composition of the oral microbiota [26]. Although the precise mechanisms by which SIgA distinguishes between commensal and pathogenic microbes remain to be fully elucidated, its concentration in saliva serves as a valuable, non-invasive proxy for evaluating oral mucosal immune status and epithelial barrier integrity [26, 28].

The assembly of the oral microbiota into stable biofilms is central to maintaining host-microbe homeostasis [19]. While relevant findings are available for humans, the influences of antibiotics on the composition, structure and function of oral microbiota in pigs, and its potential link to gut microbiota, remain largely unexplored. Therefore, this study aimed to characterize the effects of antibiotic exposure, using tylosin and a chlortetracycline/sulfadiazine/penicillin combination, on porcine oral microbiota assembly during weaning. We employed 16S rRNA gene sequencing to profile microbial changes and measured salivary SIgA and extracellular ATP to assess mucosal immunity and inflammatory status. By deciphering antibiotic-induced oral dysbiosis, this work provides valuable insights for disease prevention and management in swine production.

## Materials and methods

### Animals and study design

All animal procedures were approved by the Animal Care and Use Committee of Yangzhou University (Ethical Permit No. SYXK (Su) IACUC 2021-0026). Eighteen male Meishan piglets, weaned at 21 days of age, were obtained from a larger experiment conducted by our group. They were housed individually at a pig farm in Nanjing, China, and fed a corn-soybean meal-based diet (Table 1) formulated to meet the nutritional requirements recommended by the National Research Council [29]. The nutritional composition of the feed ingredients and dietary samples was analyzed. Crude protein concentration was determined using a K9805 nitrogen analyzer (Shanghai Analytical Instruments Co., Ltd., Shanghai, China) according to the method of American Official Society of Analytical Chemists [30]. Mineral contents (P and Ca) were analyzed using 5110 ICP-OES (Agilent Technologies, Mulgrave, Australia) [31], and amino acids (lysine, methionine, and threonine) were determined by Waters HPLC Systems (Waters Corporation, MA, USA) as previously described [32]. Piglets were randomly allocated into three experimental groups (*n* = 6 per group): a control group (CON) that received no antibiotics; a tylosin group (TYL) that received tylosin at 0.044 g/kg feed, administered via a pre-mix in 100 g of corn powder; and a combination group (CSP) that received a chlortetracycline-sulfadiazine-penicillin (2:2:1) pre-mix at a dose of 0.25 g/kg of feed. The experiment lasted for 40 d, and piglets reached 61 days of age at the endpoint. Feed intake, body weight and diarrhea occurrence were routinely monitored throughout the trial. No therapeutic drugs were administrated apart from the experimental antibiotics supplied to the TYL and CSP groups.

**Table 1:** Dietary ingredient and nutrient composition (diet basis)

Ingredients, %	Con	TYL	CSP
Corn	65	64.9956	64.975
Soy bean meal	22	22	22
Wheat	5	5	5
Bran	4	4	4
Premix^1^	4	4	4
Tylosin	-	0.0044	-
CSP	-	-	0.025
Total	100.00	100.00	100.00
**Nutritional levels**			
Digestible energy^2^, Mcal/kg			
Dry matter^3^, %	91.60	91.60	91.60
Crude protein^3^, %	16.53	16.53	16.53
Crude fat^3^, %	5.47	5.47	5.47
Crude fiber^3^, %	3.46	3.46	3.46
Crude ash^3^, %	5.08	5.08	5.08
Calcium^3^, %	0.62	0.62	0.62
Total phosphorus^3^, %	0.58	0.58	0.58
Lysine^3^, %	1.000	1.000	1.000
Methionine^3^, %	0.300	0.300	0.300
Threonine^3^, %	0.680	0.680	0.680

^1^Content per kg of premix: vitamin A, 10,800 IU; vitamin D_3_, 4,000 IU; vitamin E, 40 IU; vitamin K_3_, 4 mg; vitamin B_1_, 6 mg; vitamin B_2_, 12 mg; vitamin B_6_, 6 mg; vitamin B_12_, 0.05 mg; biotin, 0.2 mg; folic acid, 2 mg; niacin, 50 mg; D-calcium pantothenate, 25 mg; Fe, 100 mg as ferrous sulfate; Cu, 17 mg as copper sulfate; Mn, 17 mg as manganese oxide; Zn, 100 mg as zinc oxide; I, 0.5 mg as potassium iodide; and Se, 0.3 mg as sodium selenite

^2^Nutrient composition values were calculated

^3^Values determined by analysis; each value is based on triplicate determinations

### Saliva sampling

Saliva samples were collected at 0900 before morning feeding on 21, 28, 42, and 60 days of age, following a previously described method [33]. Briefly, a sterile 100% cotton rope (2–3 cm in diameter) was used. To prevent contamination, the rope was secured to the top of the cage with the free end of the rope suspended and loosened to expand the chewing area using one rope per piglet. Piglets were allowed to chew the rope for a timed period of 5 min to obtain saliva. The saturated rope was then manually squeezed into sterile centrifuge tubes pre-filled with a stabilization buffer (Beyotime Biotech, Shanghai, China). All samples were placed on ice and transported immediately to the laboratory. Upon arrival, samples were centrifuged at 3,000 × *g* for 8 min. The resulting supernatant was carefully transferred to sterile 2-mL tubes and stored at −80 °C until subsequent analysis [34].

### Blood, tissue and fecal sample collection

**Fig. 1:**
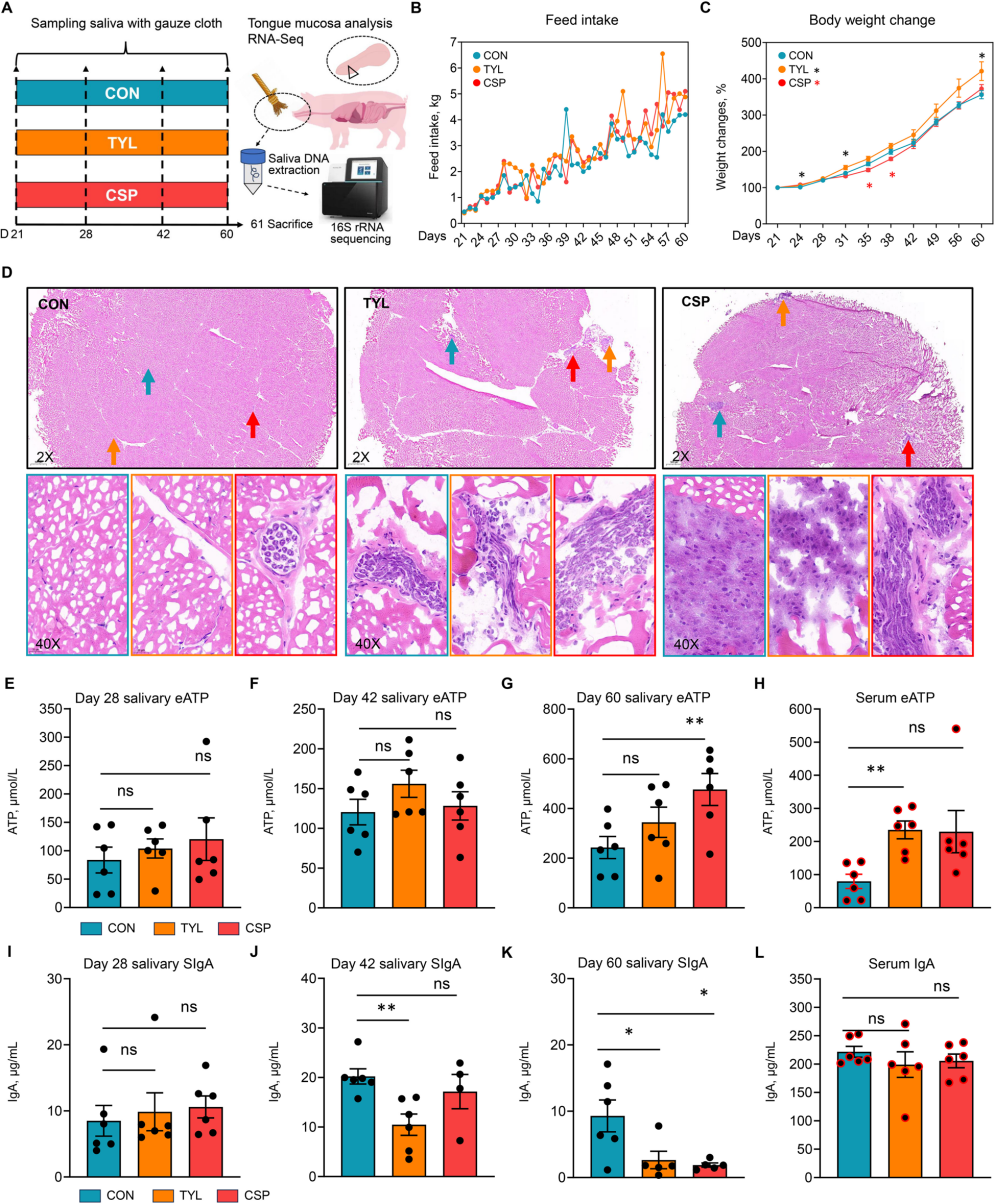
Antibiotic exposure alters oral tissue homeostasis, IgA and inflammatory responses in weaning piglets. **A** Experimental design: 21-day-old piglets were randomly allocated into three groups: a control group (CON) receiving no antibiotics, a TYL group administered tylosin, and a CSP group given a chlortetracycline-sulfadiazine-penicillin (2:2:1) premix. Antibiotic exposure lasted continuously from d 21 to d 60 of age. Saliva samples were collected at baseline (d 21) and at three subsequent time points (d 28, 42, and 60) for 16S rRNA gene amplicon sequencing and downstream analyses. All piglets were euthanized on the morning of d 61, following completion of the 40-d experimental treatment period. **B** Feed intake (kg) over the experimental period. **C** Body weight change (%) over the experimental period. **D** Representative images of H&E-stained tongue mucosa from CON, TYL, and CSP piglets at 2 × (upper panel) and 40 × (lower panel) magnification. Blue, orange, and red arrows mark the regions selected for 40 × view from the 2 × images, respectively. **E–G** Salivary extracellular adenosine triphosphate (eATP) concentrations (μmol/L) at d 28 (E), d 42 (F), and d 60 (G). **H** Serum eATP concentration (μmol/L) at the experimental endpoint. **I–K** Salivary SIgA concentrations (μg/mL) at d 28 (**I**), d 42 (**J**), and d 60 (**K**). **L** Serum IgA concentration (μg/mL) at the experimental endpoint. Data are presented as means ± SEM, with *n* = 4–6 piglets per group per time point; each dot represents an individual animal. Repeated-measures ANOVA was used for statistical analysis of feed intake and body weight, followed by post hoc multiple comparisons. One-way ANOVA with Tukey’s post hoc test was applied for eATP and IgA measurements. ns, not significant; ^*^P < 0.05; ^**^P < 0.01

At the end of the experiment, blood samples were collected from the jugular vein of each piglet. The samples were allowed to clot at room temperature for 20 min and centrifuged at 1,500 × *g* for 15 min to obtain serum. The supernatant (serum) was transferred into sterile cryovials, snap-frozen in liquid nitrogen, and stored at −80 °C for later analysis. All animals were then euthanized by exsanguination under sodium pentobarbital anesthesia. Fecal samples were collected, snap-frozen in liquid nitrogen, and stored at −80 °C. Tongue mucosa sampling was performed immediately after euthanasia on d 61. A standardized section of the dorsal lingual mucosa (anterior region, as shown in Fig. 1A) was collected from all animals in all groups. The sampled tissue was subdivided: one portion was immediately snap-frozen in liquid nitrogen and stored at −80 °C, while the other was processed into sections for morphological examination.

### Serum biochemical analysis

Serum levels of albumin (ALB), total protein (TP), triglycerides (TG), non-esterified fatty acids (NEFA), total cholesterol (TCHO), high-density lipoprotein cholesterol (HDLC), low-density lipoprotein cholesterol (LDLC), iron (Fe), glucose (GLU), uric acid (UA), blood urea nitrogen (BUN), alanine aminotransferase (ALT), aspartate aminotransferase (AST), alkaline phosphatase (ALP), lactate dehydrogenase (LDH), and immunoglobulins (IgA, IgG, and IgM) were analyzed using a fully automated biochemical analyzer (KHB 450, Shanghai Kehua Bioengineering, Shanghai, China) with matched commercial reagents according to the manufacture’s instruction.

### Enzyme-Linked Immunosorbent Assay (ELISA)

Salivary SIgA levels were quantified using an ELISA kit (CSB-E12063p, Cusabio Biotech, Wuhan, China) following the manufacturer’s instructions. Briefly, samples were diluted in PBS containing protease inhibitors (Beyotime, Nanjing, China) at a final concentration of 0.1 mg/µL, gently vortexed, and centrifuged at 400 × *g* for 5 min to remove food residues and particulate matter. The supernatant was subsequently filtered through a sterile 70 µm cell strainer and centrifuged at 8,000 × *g* for 10 min to separate bacterial cells. The resulting supernatant was transferred to a new sterile tube and further centrifuged at 19,000 × *g* for 10 min to eliminate fine particles. The final clarified supernatant was used for ELISA-based quantification of SIgA.

### Measurement of ATP

Extracellular adenosine triphosphate (eATP) levels in saliva were measured as an indicator of inflammatory responses [35, 36] using a fluorescing-luciferase based ATP assay kit (S0026B, Beyotime Biotech, Shanghai, China) according to the manufacturer’s instructions. Briefly, the samples were weighed and suspended in PBS containing 0.01% NaN_3_, and protease inhibitors at a concentration of 0.1 mg/µL. The homogenate was centrifuged at 400 × *g* for 5 min in a pre-cooled centrifuge to remove large debris, and the supernatant was collected, further centrifuged at 8,000 × *g* for 10 min, filtered through a 0.22-µm sterile filter. Finally, the filtrate was used to measure ATP levels.

### Histological analysis

For histological analysis, tissue samples from the anterior tongue were dissected out and fixed in 4% paraformaldehyde, processed, embedded in paraffin, and sectioned at a thickness of 4 µm. Sections were then stained with hematoxylin and eosin (H&E) following the manufacturer's protocol for morphological evaluation (G1120, Solarbio, China). All stained sections were examined and photographed under a light microscope (Leica DMi8, Leica, Wetzlar, Germany).

### RNA extraction and transcriptomic analysis

Total RNA was extracted from tissue samples using TRIzol Reagent (Invitrogen, 15596026, Waltham, MA, USA) and quantified with a NanoDrop 1000 spectrophotometer. RNA quality was assessed using an Agilent Bioanalyzer 2100 system (Agilent Technologies, CA, USA). RNA-seq libraries were constructed using the Illumina TruSeq RNA Sample Prep Kit (Illumina, CA, USA), and sequenced on the Illumina HiSeq 2000 platform at BGI Tech (Wuhan, China). Raw sequencing data were filtered using SOAPnuke (v1.5.6) to o generate clean reads in FASTQ format. High-quality clean reads were aligned to the *Sus scrofa* reference genome (Sscrofa11.1) along with its corresponding annotation files using Bowtie2 (v2.5.0). Gene expression levels were quantified as fragments per kilobase of exon per million mapped reads (FPKM) using Cufflinks. Differential gene expression analysis was performed via DESeq2. Genes with |log_2_ (fold change)| > 1 and an adjusted *P*-value < 0.05 were defined as differentially expressed genes.

### Saliva DNA extraction

DNA was extracted from saliva samples using the MagPure Stool DNA KF Kit B (MAGEN, Guangzhou, China) according to the manufacturer's instructions. Briefly, 100–200 mg of each sample was transferred to a centrifuge tube containing grinding beads. One mL of buffer ATL/PVP-10 was added, and the sample was homogenized using a grinding machine (Shanghai Jingxin Tech, China) and incubated at 65 °C for 20 min. The mixture was centrifuged at 14,000 × *g* for 5 min (Eppendorf, German). The supernatant was transferred to a new tube, and 0.6 mL of buffer PCI was added. The solution was mixed thoroughly by vortexing for 15 s and then centrifuged at 18,213 × *g* for 10 min. The supernatant was transferred to a deep-well plate containing a prepared magnetic bead binding solution (600 Buffer with magnetic beads, 20 Proteinase K, and 5 μL RNase A). The plate was then processed on a Kingfisher system (Thermo Fisher Scientific, Waltham, MA, USA) using the appropriate program for automated extraction, which included three washes with 700 μL of washing buffers. Finally, DNA was eluted in 100 μL of elution buffer and collected in a 1.5-mL centrifuge tube.

### 16S rRNA gene amplification and sequencing

The library was prepared using 2 × Phanta Max Master Mix (VAZYME, China) and the V4 region of 16S rDNA was amplified using forward and reverse PCR primers (515F: 5′-GTGCCAGCMGCCGCGGTAA-3′, 806R: 5′-GGACTACHVGGGTWTCTAAT-3′). PCR was performed in a 50 μL reaction with 30 ng of template DNA and fusion primers. The cycling conditions were: 95 °C for 3 min; 30 cycles of 95 °C for 15 s, 56 °C for 15 s, and 72 °C for 45 s, followed by a final extension at 72 °C for 5 min. PCR products were purified using DNA magnetic beads (BGI, LB00V60). The validated libraries were sequenced on the Illumina MiSeq platform (BGI, Shenzhen, China), generating 2 × 250 bp paired-end reads following standard Illumina protocols. Primers, low-quality sequences, and barcode sequences were removed from the raw reads. The contralateral clean reads were merged using FLASH (Baltimore, MD, USA, version 1.2.11) with a minimum overlap of 10 bp and a mismatch error rate of 2%. Operational taxonomic unit (OTU) with 97% similarity were identified using the UPARSE (version 9.2.64) pipeline. Bacterial diversity was estimated for the observed species, Shannon, and Simpson indexes using the MOTHUR program (version 1.35.0). Microbial gene function was predicted using the PICRUSt (version 2.1.4) software and annotated using the Kyoto Encyclopedia of Genes and Genomes (KEGG) database [37, 38].

### Bacteria quantification by RT‑qPCR analysis

The abundance of bacterial DNA was quantified using previously validated qPCR primers (Supplementary Table S1). For specific bacteria quantification, qPCR reactions were conducted in a final volume of 10 μL, containing 1 × SYBR Green Master Mix (Qiagen GmbH, Hilden, Germany), 0.3 μmol/L of each primer, and 12.5 ng of DNA template. The thermal cycling program was set as follows: initial denaturation 95 °C for 5 min, 40 cycles of 95 °C for 15, 40 and 30 s; followed by a final extension step at 72 °C for 5 min. Nuclease-free water was used as the no-template negative control. For universal eubacterial quantification of total bacterial load, an independent TaqMan-based qPCR assay was applied with universal 16S rDNA primers and a 0.25 µmol/L FAM-labeled probe: 6-FAM-5′-CGTATTACCGCGGCTGCTGGCAC-3′-TAMRA, which targets the conserved region of the bacterial 16S rRNA gene [39]. This assay was performed using TaqMan Universal PCR Master Mix (Applied Biosystems). All amplifications were carried out on a 7500 Real-Time PCR System (Thermo Fisher Scientific, Waltham, MA, USA). Fluorescence signals were collected via the SYBR channel for species-specific assays and the FAM channel for total bacterial quantification. All samples were tested in technical duplicates to guarantee experimental reproducibility. Each qPCR plate included a plasmid DNA standard curve (triplicate) to convert threshold cycle (Ct) values into copy numbers per microliter of template. The DNA concentration for both samples and standards was maintained at 12.5 ng per reaction. Standard curves yielded *R*^2^ values of 0.990 ± 0.009, and the detection limit was established at 500 copies. To normalize data distribution, bacterial abundance was calculated based on the logarithmic copy number of the 16S rRNA gene.

### Statistical analysis

Statistical analyses were performed using GraphPad Prism 9 (GraphPad Software, Inc, CA, USA) and R 4.3.3. Repeated-measures analysis of variance (ANOVA) was adopted to assess longitudinal variations in feed intake and body weight, accounting for correlated repeated measurements from the same individual. For normally distributed continuous indicators, two-tailed independent *t*-test was applied for pairwise comparison, while one-way ANOVA combined with Tukey’s post hoc test was used for multigroup comparisons. Microbiota OTU profiles were processed on the Omicshare platform (www.omicshare.com). The Bray–Curtis distance matrix was calculated, and principal coordinate analysis (PCoA) was performed and visualized using the ggplot2 package in R. Venn diagrams were generated using online tools from OmicShare and Bioinformatics Cloud (http://www.bioinformatics.com.cn). Orotype classification was performed on the Majorbio Cloud Platform (https://www.majorbio.com/tools) using OTU level data. Functional pathway abundance differences were annotated against the KEGG database and analyzed using STAMP software with Welch’s *t*-test and Benjamini–Hochberg FDR correction. Microbial α-diversity was displayed as box plots presenting median, interquartile range, minimum and maximum values. Other quantitative data are presented as mean ± SEM. The sample size (*n*) of each group is specified in figure legends. A *P*-value < 0.05 was considered statistically significant.

## Results

### Antibiotic exposure during weaning impairs tongue mucosa integrity, suppresses IgA production and induces inflammatory responses in piglets

Early establishment of oral biofilm and commensal microbiota sustains host-microbiota homeostasis, while disruption of this balance compromises physiological function [19]. Here, we performed 16S rRNA gene sequencing to characterize saliva microbiota of weaned piglets at 21, 28, 42 and 60 days of age, alongside physiological measurements, to explore the impacts of early antibiotic perturbation during the weaning period (Fig. 1A). No significant differences in feed intake were observed across the CON, TYL and CSP groups (Fig. 1B, *P* > 0.05). In contrast, piglets in the TYL group exhibited significantly greater relative weight gain than the CON group on d 24, 31, and 60 (*P* < 0.05). The CSP group showed significantly lower relative weight gain compared to the CON group on d 35 and 38 (*P* < 0.05), whereas this discrepancy disappeared at d 60 (*P* > 0.05). Furthermore, porcine tongue mucosal morphology was evaluated via H&E staining as previously described [40, 41]. It revealed well-preserved lingual architecture in the CON group, with intact stratified squamous epithelium, orderly muscle bundles, and no inflammation (Fig. 1D, left). In contrast, the TYL group showed mild focal inflammation, including perivascular inflammatory cuffing and scattered neutrophils in the interstitium and muscle fascicles (middle). The CSP group also displayed low-grade inflammation, with sparse neutrophils in vascular lumina and extravascular compartments (right). No severe lesions, necrosis, or extensive infiltrates were observed in either treatment group, that muscular architecture and mucosal integrity remained largely intact across all groups.

Measurement of salivary eATP levels showed no significant changes in the TYL group during weaning (Fig. 1E–G, *P* > 0.05). In the CSP group, eATP levels were unchanged at d 28 and 42 of age (*P* > 0.05) but increased significantly compared to the CON group by d 60 of age (Fig. 1G, *P* < 0.01). Notably, serum eATP levels were significantly elevated in the TYL group compared to the CON group (Fig. 1H, *P* < 0.01), indicating a systemic inflammatory response. No significant difference was observed between the CSP and CON groups (*P* > 0.05). In addition, serum biochemical parameters were measured to evaluate the effects of antibiotics on nutrient metabolism and physiological status of piglets (Table 2). Compared with the control group, piglets in the TYL and the CSP groups showed significantly decreased levels of ALB, TP, GLU, serum iron, multiple lipid metabolites, and circulating immunoglobulins (*P* < 0.05). The activities of metabolic enzymes, including ALT, ALP, and LDH were also significantly altered (*P* < 0.05).

**Table 2:** The effects of antibiotic exposure on serum biochemical parameters of piglets at the experimental endpoint

Parameter	Unit	CON	TYL	CSP
ALB	g/L	27.165 ± 1.630^a^	12.974 ± 1.049^b^	16.161 ± 1.31^b^
TG	mmol/L	0.131 ± 0.008^a^	0.047 ± 0.006^b^	0.090 ± 0.018^ab^
NEFA	mmol/L	0.200 ± 0.002^a^	0.169 ± 0.006^b^	0.163 ± 0.004^b^
TCHO	mmol/L	2.271 ± 0.225^a^	0.553 ± 0.118^b^	0.330 ± 0.038^b^
Fe	μmol/L	9.327 ± 0.074^a^	8.554 ± 0.258^b^	8.591 ± 0.171^b^
GLU	mmol/L	6.971 ± 0.134^a^	3.793 ± 0.590^b^	2.750 ± 0.488^b^
UA	μmol/L	20.688 ± 0.598^a^	15.644 ± 1.566^b^	20.351 ± 1.067^a^
ALT	U/L	13.006 ± 0.376^a^	10.141 ± 0.161^b^	10.032 ± 0.104^b^
AST	U/L	23.456 ± 0.840^a^	24.117 ± 0.528^a^	23.633 ± 0.409^a^
ALP	U/L	69.727 ± 0.191^a^	65.644 ± 0.538^b^	65.734 ± 0.483^b^
BUN	mmol/L	3.362 ± 0.078^a^	3.462 ± 0.203^a^	2.399 ± 0.450^a^
TP	g/L	56.067 ± 0.559^a^	30.723 ± 1.307^b^	31.174 ± 6.052^b^
HDLC	mmol/L	1.096 ± 0.020^a^	0.628 ± 0.182^b^	0.809 ± 0.234^b^
LDH	U/L	246.41 ± 10.571^a^	182.40 ± 18.678^b^	169.86 ± 13.666^b^
LDLC	mmol/L	1.642 ± 0.065^a^	0.638 ± 0.148^b^	0.352 ± 0.067^b^
IgA	g/L	0.240 ± 0.006^a^	0.225 ± 0.011^a^	0.216 ± 0.010^a^
IgG	g/L	1.022 ± 0.005^a^	0.823 ± 0.068^ab^	0.621 ± 0.146^b^
IgM	g/L	1.222 ± 0.066^a^	0.712 ± 0.054^b^	0.561 ± 0.037^b^

*ALB* Albumin, *TP* Total protein, *TG* Triglycerides, *NEFA* Non-esterified fatty acids, *TCHO* Total cholesterol, *HDLC* High-density lipoprotein cholesterol, *LDLC* Low-density lipoprotein cholesterol, *Fe* Iron, *GLU* Glucose, *UA* Uric acid, *BUN* Blood urea nitrogen, *ALT* Alanine aminotransferase, *AST* Aspartate aminotransferase, *ALP* Alkaline phosphatase, *LDH* Lactate dehydrogenase, *IgA* Immunoglobulin A, *IgG* Immunoglobulin G, *IgM* Immunoglobulin M

^a,b^Values in the same row without a common superscript differ significantly (*P* < 0.05)

Given that salivary SIgA serves as a frontline of immune defense in controlling the oral microbiota [19, 42], we quantified its levels. No significant difference was detected in the first week post-weaning (Fig. 1I, *P* > 0.05). However, by d 42, salivary SIgA levels in the TYL group were significantly lower than in the CON group (Fig. 1J, *P* < 0.01). This suppression became more pronounced by d 60, with both the TYL and CSP groups exhibiting significantly reduced salivary SIgA levels compared to controls (Fig. 1K, *P* < 0.05). In contrast, no significant difference was observed in serum IgA levels between the TYL or CSP groups and the CON group at the end point of the experiment (Fig. 1L, *P* > 0.05).

Collectively, these data indicate that low-dose antibiotic exposure during weaning suppresses overall metabolic activity and compromises mucosal immune responses in oral cavity of piglets.

### Antibiotic exposure during weaning alters transcriptomic program of tongue mucosa in piglets

**Fig. 2:**
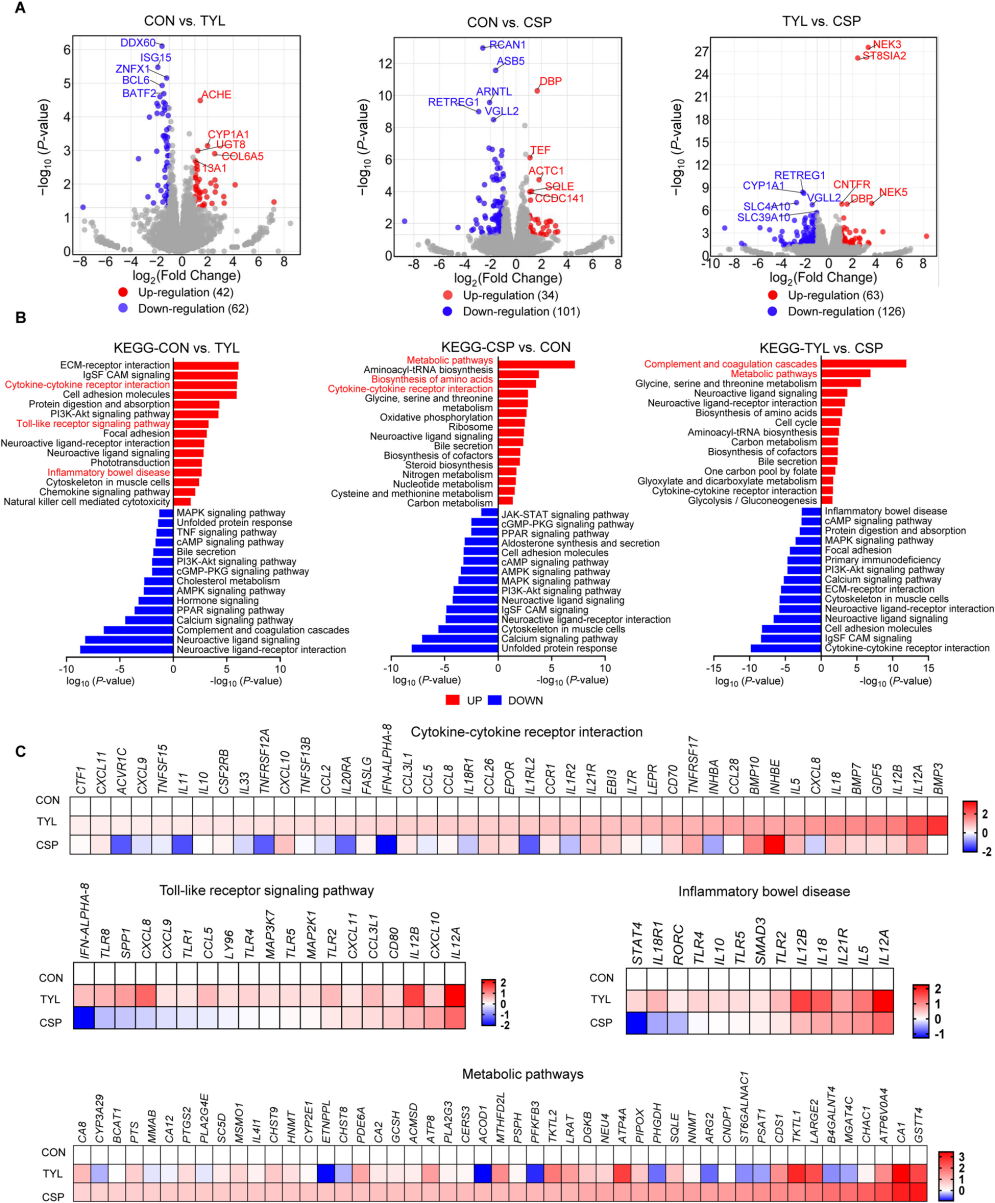
Antibiotic exposure alters transcriptomic program of tongue mucosa in weaning piglets. **A** The dorsal lingual mucosa (one side) was harvested with scissors for RNA-Seq. Volcano plot visualization of the differentially expressed genes with top five genes labeled. **B** Enrichment pathways were analyzed using KEGG for the comparisons between TYL vs. CON, CSP vs. CON, and TYL vs. CSP. **C** Heatmaps depicting the enrichment of genes up- or down-regulated in the “Cytokine-cytokine receptor interaction”, “Toll-like receptor signaling pathway”, “Inflammatory bowel disease” and “Metabolic pathways”. A hypergeometric test with Benjamini–Hochberg false discovery rate (FDR) correction was applied

To delineate the effects of antibiotic exposure on the oral niche where microbiota reside, we performed RNA-Seq analysis on anterior tongue tissues from piglets. Volcano plot analysis identified 42 upregulated and 62 downregulated genes in the TYL vs. CON comparison. The most strongly downregulated genes included *DDX60, ISG15, ZNFX1, BCL6*, and *BATF2*, while *ACHE*, *CYP1A1*, *UGT8*, *COL6A5*, and *ITGA1* were among the most significantly upregulated genes. In the CSP vs. CON comparison, 34 genes were upregulated and 101 genes were downregulated, with *RCAN1*, *ASB5*, *ARNTL*, *RETREG1*, and *VGLL2* identified as the top downregulated genes, whereas *DBP*, *TEF*, *ACTC1*, *SQLE*, and *CCDC141* were the top upregulated genes. For the TYL vs. CSP comparison, 63 upregulated and 126 downregulated genes were detected, with *RETREG1*, *CYP1A1*, *SLC4A10*, *SLC39A10*, and *VGLL2* as the most significantly downregulated, and *NEK3, ST8SIA2, CNTFR, DBP,* and *NEK5* as the most strongly upregulated genes. For the TYL vs. CSP comparison, 63 upregulated and 126 downregulated genes were detected, with *RETREG1*, *CYP1A1*, *SLC4A10, SLC39A10*, and *VGLL2* as the most significantly downregulated, and *NEK3*, *ST8SIA2*, *CNTFR*, *DBP*, and *NEK5* as the most strongly upregulated genes (Fig. 2A). Gene ontology enrichment analysis of KEGG pathways showed that TYL exposure upregulated pathways linked to immune response (e.g., “Cytokine-cytokine receptor interaction” and “Toll-like receptor signaling pathway”) and inflammation (e.g., “Inflammatory bowel disease”). In contrast, CSP exposure upregulated "Metabolic pathways" and "Biosynthesis of amino acids", in addition to "Cytokine-cytokine receptor interaction". Meanwhile, "Complement and coagulation cascades" and "Metabolic pathways" were among the top enriched pathways in CSP compared to TYL (Fig. 2B).

Consistent with these findings, heatmap analysis revealed antibiotic-driven upregulation of specific genes: *CTF1*, *CXCL11*, *ACVR1C*, *CXCL9*, *TNFSF15*, *IL1*, *IL10*, *CSF2RB*, *IL33*, *TNFRSF12A*, *CXCL10*, *TNFSF13B*, *CCL2*, *IL20RA*, *FASLG*, *IFN-ALPHA-8*, *CCL3L1*, *CCL5*, *CCL8*, *IL19R1*, *CCL26*, *EPOR*, *IL1RL2*, *CCR1*, *IL1R2*, *IL21R*, *EB13*, *IL7R*, *LEPR*, *CD70*, *TNFSF17*, *INHBA*, *CCL28*, *BMP10*, *INHBE*, *IL5*, *CXCL8*, *IL18*, *GDF5*, *BMP7*, *IL12B*, *IL12A*, and *BMP3* from the ‘Cytokine-cytokine receptor interaction’; *IFN-ALPHA-8*, *TLR8*, *SPP1*, *CXCL8*, *CCL5*, *TLR4*, *LY96*, *TLR9*, *MAP3K7*, *TLR5*, *MAP2K1*, *TLR2*, *CXCL11*, *CCL3L1*, *CD80*, *CXCL10*, *IL12B*, and *IL12A* from the ‘Toll-like receptor signaling pathway’, and *STAT4*, *IL18R1*, *RORC*, *TLR4*, *TLR5*, *IL10, SMAD3*, *TLR2*, *IL12B*, *IL18*, *IL21R*, *IL5*, and *IL12A* from ‘Inflammatory bowel disease’ (Fig. 2C, upper and middle panels). In addition, antibiotic exposure resulted in the upregulation of genes from "Metabolic pathways", including *CA8*, *CYP3A29*, *BCAT1*, *MMAB*, *CA12*, *PTGS2*, *PLA2G4E*, *SC5D*, *MSMO1*, *IL4I1*, *CHST9*, *HNMT*, *CYP2E1*, *ETNPPL*, *CHST8*, *PDE4A*, *CA2*, *GCSH*, *ACMSD*, *PLA2G3*, *ATP8*, *CERS3*, *ACOD1*, *MTHFD2L*, *PSPH*, *FKFKB3*, *TKT1*, *LRAT*, *NEU4*, *DGKB*, *ATP4A*, *PIPOX*, *PHGDH*, *SQLE*, *NAGT2*, *ARNT2*, *CNDP1*, *ST6GALNAC1*, *PSAT1*, *DS1*, *LARGE2*, *B4GALNT4*, *MGAT4C*, *CHAC1*, *ATP6V0A4*, *CA1*, and *GSTT4* (Fig. 2C, bottom panel). These data indicate that antibiotic exposure exhibited certain common ways in regulating host physiology, but with distinctive focuses. In particular, antibiotic exposure converges on the dysregulation of immune and inflammatory pathways, with CSP specifically impacting metabolic reprogramming, which may further amplify the shared immune response.

### Antibiotic exposure during weaning disrupts the establishment of the orotype and induces microbiota imbalance

**Fig. 3:**
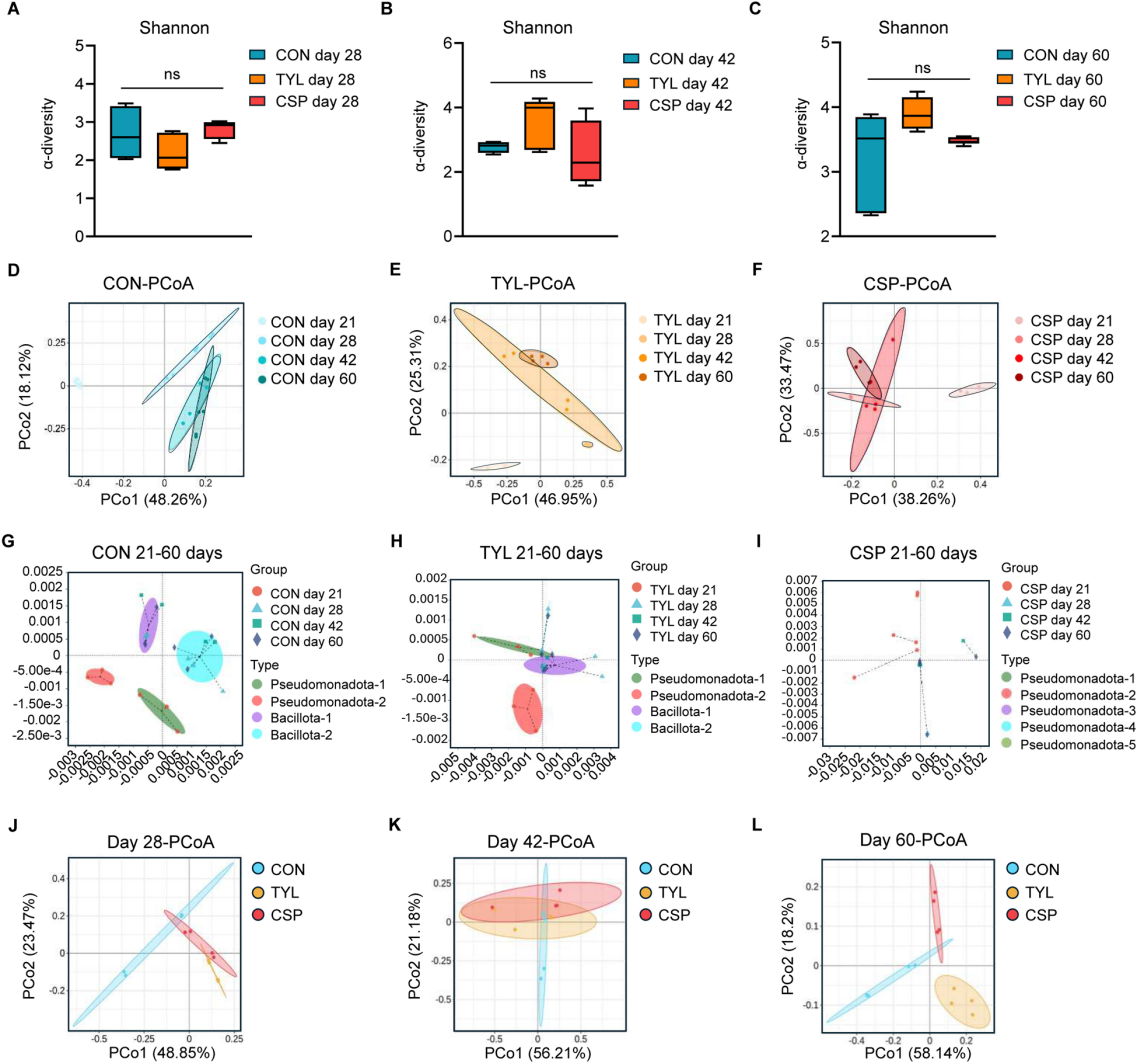
Antibiotic exposure disrupts the establishment of the oral microbiota in weaning piglets. 16S rRNA gene amplicon sequencing was performed to characterize the oral microbiota of piglets in the CON, TYL, and CSP groups at d 21, 28, 42, and 60 of age. **A**–**C** Shannon index at d 28 (**A**), d 42 (**B**), and d 60 (**C**) was calculated to evaluate α-diversity of the microbial community. Data are presented as box plots showing the median, interquartile range (IQR), and minimum/maximum values for each group and were determined using one-way ANOVA with Tukey’s post hoc test. ns, not significant. **D**–**F** Principal coordinate analysis (PCoA) based on Bray–Curtis distance matrices show the temporal trajectory of the oral microbiota within each group: CON (**D**), TYL (**E**), and CSP (**F**). **G**–**I** Clustering analysis using an abundance-based distance algorithm revealed the oral microbial community structure (referred to as orotypes) in the CON (**G**), TYL (**H**), and CSP (**I**) groups across time points. **J**–**L** Between-group comparisons of oral microbiota using PCoA at d 28 (**J**), d 42 (**K**), and d 60 (**L**) are shown. *n* = 4–6 piglets per group per time point; each dot represents an individual animal

We next evaluated the impact of antibiotic exposure on the oral microbiota of weanling piglets using 16S rRNA gene sequencing, which yielded a total of 11,458,000 raw reads across all samples, with an average of 169,093 ± 97 reads per sample, ensuring sufficient sequencing depth for downstream microbiota analysis. Alpha diversity, as measured by Shannon index, showed no significant differences among groups at 28, 42, or 60 days of age (Fig. 3A–C, *P* > 0.05). We then analyzed temporal shifts in microbial community structure using principal coordinate analysis (PCoA; Fig. 3D–F). In control piglets, the oral microbiota at d 21 formed a distinct cluster separated from all post-weaning time points along PCo1 (48.26%). Microbial profiles at d 28 exhibited partial overlap with later time points, while d 42 and 60 d communities clustered closely together, indicating stabilization of the oral microbiota by d 42 (Fig. 3D). In the TYL group, d 21 microbiota also formed a unique cluster (PCo1, 46.95%), with subsequent time points (d 28, 42, and 60 d) showing extensive overlap (Fig. 3E). Similarly, in the CSP group, d 21 microbiota were clearly distinct (PCo1, 38.26%), with no obvious separation observed among d 28, 42, and 60 (Fig. 3F). These temporal dynamics were further supported by orotype analysis (Fig. 3G–I), defined as oral microbial community stratification [43], a concept adapted from enterotyping, which identifies highly populated regions in the multidimensional space of community organization [44]. In controls, two dominant orotypes were identified: an early Pseudomonadota-dominated type (driven primarily by Moraxellaceae) at d 21, which shifted to a Bacillota-dominated type (driven by Lachnospiraceae) from d 28 onward, with each orotype subdivided into two distinct sub-clusters (Fig. 3G). In the TYL group, the initial orotype at d 21 was also Pseudomonadota (Moraxellaceae)-enriched. From d 28 onward, the community transitioned to a more dispersed Bacillota-dominated orotype shaped by a consortium of taxa including Veillonellaceae, Lachnospiraceae, Prevotellaceae, and Streptococcaceae (Fig. 3H). In contrast, the CSP group retained a Pseudomonadota-dominated orotype throughout the weaning period, while this orotype was initially driven by both Moraxellaceae and Pasteurellaceae at d 21, it failed to transition to the Bacillota-dominated orotype seen in control piglets (Fig. 3I).

We further compared microbial community structure among groups at each post-weaning time point. PCoA at d 28 (PCo1, 48.85%; PCo2, 23.47%), d 42 (PCo1, 56.21%; PCo2, 21.18%), and d 60 (PCo1, 58.14%; PCo2, 18.2%) revealed distinct clustering of CON, TYL, and CSP groups (Fig. 3J–L), indicating persistent antibiotic-induced alterations in oral microbiota composition throughout weaning.

**Fig. 4:**
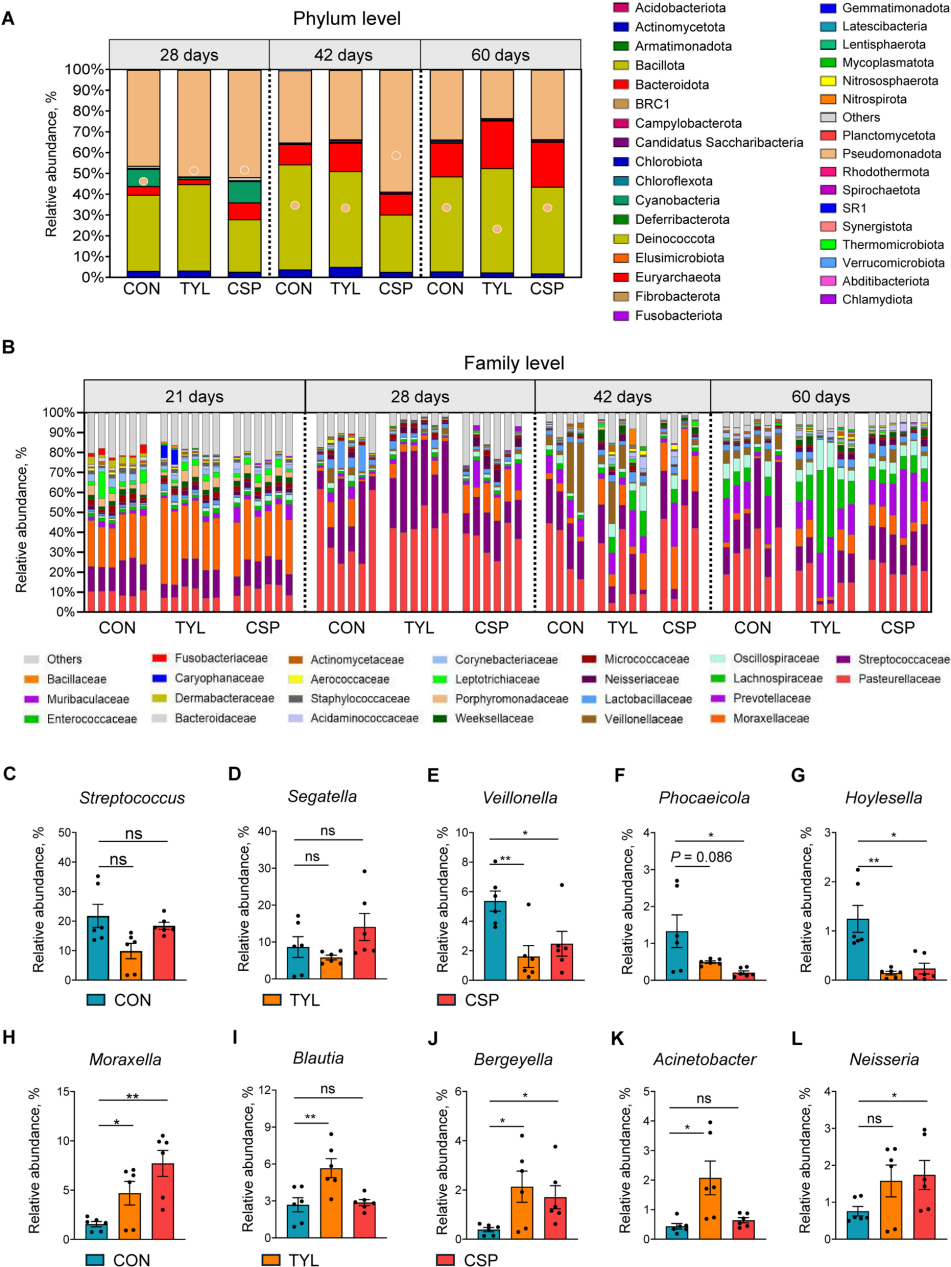
Antibiotic exposure induces microbiota imbalance at various taxonomic levels in the oral cavity of weaning piglets. Oral microbiota composition was analyzed at the phylum (**A**) and family (**B**) levels in the CON, TYL, and CSP groups at 21, 28, 42, and 60 days of age. **C**–**L** Relative abundance of *Streptococcus*, *Segatella, Veillonella, Phocaeicola*, *Hoylesella*, *Moraxella*, *Blautia*, *Bergeyella*, *Acinetobacter*, *Neisseria* in the oral microbiota of the CON, TYL, and CSP groups. Data are presented as the means ± SEM, *n* = 4–6 samples per group per time point; each dot represents an individual animal. Statistical significance was assessed by one-way ANOVA followed by Tukey’s post hoc test; ns, not significant; ^*^P < 0.05, ^**^P < 0.01

The effects of antibiotic exposure on oral microbial composition were also evident at the phylum, family and dominant genus levels (Fig. 4). At the phylum level, Pseudomonadota, Bacillota, Bacteroidota, Actinomycetota and Cyanobacteriota were predominant, with no significant differences between the CON and antibiotic-treated groups after weaning (Fig. 4A, *P* > 0.05). At the family level, Streptococcaceae, Pasteurellaceae, Prevotellaceae, and Moraxellaceae dominated the community, and no significant differences were observed among the CON, TYL and CSP groups at baseline on d 21 (Fig. 4B). On d 28, both TYL and CSP significantly reduced the relative abundance of Lactobacillaceae (*P* < 0.05). By d 42, both treatments significantly increased Moraxellaceae and decreased Veillonellaceae (*P* < 0.05). On d 60, TYL significantly reduced Streptococcaceae (*P* < 0.05), while CSP significantly increased Lactobacillaceae (*P* < 0.05). Furthermore, at this final time point, both antibiotics reduced Pasteurellaceae and Veillonellaceae (*P* < 0.05) while increasing Moraxellaceae (*P* < 0.05).

At the genus level by d 60, no significant difference was observed in the relative abundance of *Streptococcus* (Fig. 4C, *P* > 0.05) or *Segatella* (Fig. 4D, *P* > 0.05) among groups. In contrast, TYL administration significantly reduced the relative abundance of *Veillonella* (Fig. 4E, *P* < 0.05) and *Hoylesella* (Fig. 4G, *P* < 0.01), while increasing *Moraxella* (Fig. 4H, *P* < 0.05), *Blautia* (Fig. 4I, *P* < 0.01), *Bergeyella* (Fig. 4J, *P* < 0.05), and *Acinetobacter* (Fig. 4K, *P* < 0.05). CSP significantly reduced *Veillonella* (Fig. 4E, *P* < 0.05), *Phocaeicola* (Fig. 4F, *P* < 0.05), and *Hoylesella* (Fig. 4G, *P* < 0.05), while increasing *Moraxella* (Fig. 4H, *P* < 0.05), *Bergeyella* (Fig. 4J, *P* < 0.05), and *Neisseria* (Fig. 4L, *P* < 0.01).

**Fig. 5:**
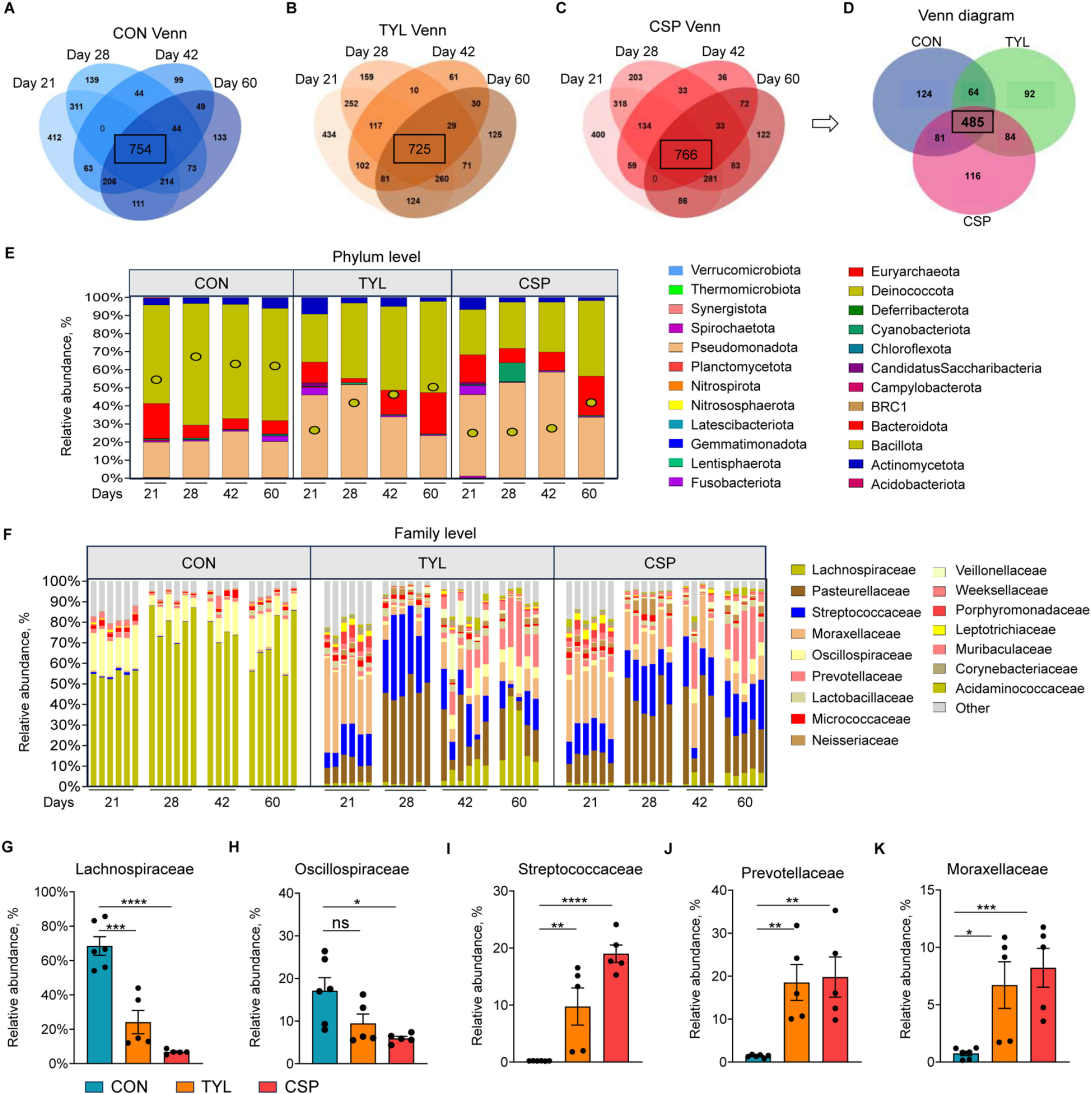
The core microbiota in the oral cavity resistant to antibiotic exposure and stabilize during weaning in piglets. **A**–**C** Venn diagrams showing the shared microbiota during weaning through time in piglets from the CON, TYL and CSP groups, respectively. Each ellipse represents a specific time, and the overlapping regions between ellipses represent shared OTUs within groups. **D** Venn diagram illustrating the overlap of temporally persistent core microbial taxa across the CON, TYL and CSP groups. **E** and **F** Bar graphs showing the composition of temporally stable core microbiota at the phylum (**E**) and family (**F**) levels, derived from the temporal Venn analyses in panels (**A**–**C**). **G**–**K** Relative abundance of Lachnospiraceae, Oscillospiraceae, Streptococcaceae, Prevotellaceae and Moraxellaceae in oral microbiota from the CON, TYL and CSP groups. Data are presented as the means ± SEM, *n* = 4–6 samples per group per time point, each dot represents an individual animal. ^ns^*P* > 0.05, ^*^P < 0.05, ^**^P < 0.01, ^***^P < 0.001, ^****^P < 0.0001, using ANOVA with Tukey’s post hoc test

Finally, we characterized the temporally stable oral microbiota within each group to identify keystone bacteria resistant to perturbation during weaning. Venn diagrams revealed 754, 725 and 766 shared OTUs across all time points in the CON, TYL, and CSP groups, respectively (Fig. 5A–C). Furthermore, we constructed a cross-group Venn diagram of the temporally stable core microbiota (Fig. 5D). The analysis showed that 485 OTUs were common to all three groups. The CON group contained 124 unique core OTUs, while TYL and CSP had 92 and 116 unique core OTUs, respectively. Partial overlaps were also observed between CON-TYL (64 OTUs), CON-CSP (81 OTUs), and TYL-CSP (84 OTUs).

These core OTUs were primarily assigned to the phyla Bacillota, Bacteroidota, and Pseudomonadota (Fig. 5E). At the family level (Fig. 5F), in the CON group, Lachnospiraceae and Oscillospiraceae consistently maintained a predominant position throughout the weaning period. In contrast, the TYL and CSP groups were persistently dominated by Pasteurellaceae, Streptococcaceae, and Moraxellaceae. Notably, Prevotellaceae, which was less abundant at early time points, rose to dominance by d 60 in both antibiotic-treated groups, indicating a delayed but substantial response to low-dose antibiotic exposure.

Consistent with these compositional patterns, the CON group exhibited a higher relative abundance of the families Lachnospiraceae (Fig. 5G, *P* < 0.001) and Oscillospiraceae (Fig. 5H, *P* < 0.05) compared to the antibiotic-treated groups. In contrast, the TYL and CSP groups showed a higher abundance of Prevotellaceae (Fig. 5I, *P* < 0.01), Streptococcaceae (Fig. 5J, *P* < 0.01), Pasteurellaceae (Fig. 5K, *P* < 0.01), and Moraxellaceae (Fig. 5K, *P* < 0.05).

### Antibiotic exposure during weaning alters oral microbiota KEGG pathways and functional prediction in piglets

**Fig. 6:**
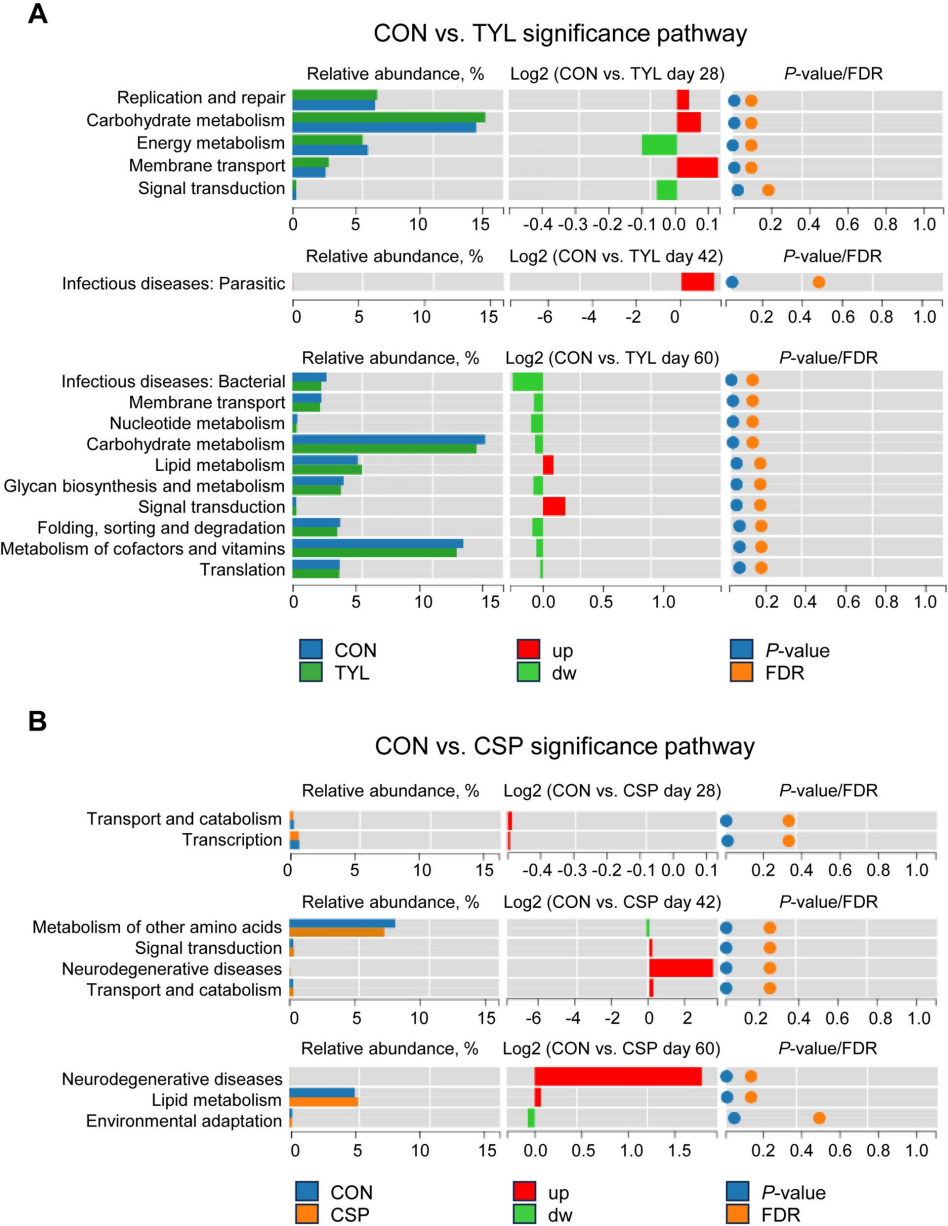
Antibiotic exposure alters oral microbiota KEGG pathways and functional prediction in weaning piglets. **A** and **B** Different groups of microbial populations were subjected to predicted KEGG pathway enrichment analysis at 28, 42 and 60 days of age

The results above suggest that antibiotic exposure during weaning induces oral microbiota dysbiosis, which likely alters its function. We therefore performed KEGG functional prediction analysis based on 16S rRNA gene sequencing data. For the TYL group at d 28, treatment significantly downregulated pathways related to energy metabolism and signal transduction (Fig. 6A, upper panel, *P* < 0.05), while upregulating pathways associated with replication and repair (*P* < 0.05), carbohydrate metabolism, and membrane transport (*P* < 0.05). By d 42, TYL significantly upregulated pathways related to infectious diseases: parasitic (Fig. 6A, middle panel, *P* < 0.05). At d 60, TYL treatment significantly reduced pathways associated with infectious diseases: bacterial (Fig. 6A, lower panel), membrane transport, nucleotide metabolism, carbohydrate metabolism, glycan biosynthesis and metabolism, folding, sorting and degradation, metabolism of cofactors and vitamins, and translation (*P* < 0.05). In contrast, it significantly upregulated lipid metabolism and signal transduction at this final time point (*P* < 0.05). For the CSP group at d 28, treatment significantly upregulated pathways associated with transport and catabolism (Fig. 6B, upper panel) and transcription (*P* < 0.05). By d 42, CSP significantly downregulated pathways related to the metabolism of other amino acids (Fig. 6B, middle panel, *P* < 0.05) while upregulating pathways for signal transduction, neurodegenerative diseases, and transport and catabolism (*P* < 0.05). By d 60, CSP significantly reduced the environmental adaptation pathway (Fig. 6B, lower panel, *P* < 0.05), and upregulated pathways related to neurodegenerative diseases and lipid metabolism (*P* < 0.05).

### Antibiotic exposure during weaning affects *Phocaeicola* in both oral and intestinal microbiota of piglets

**Fig. 7:**
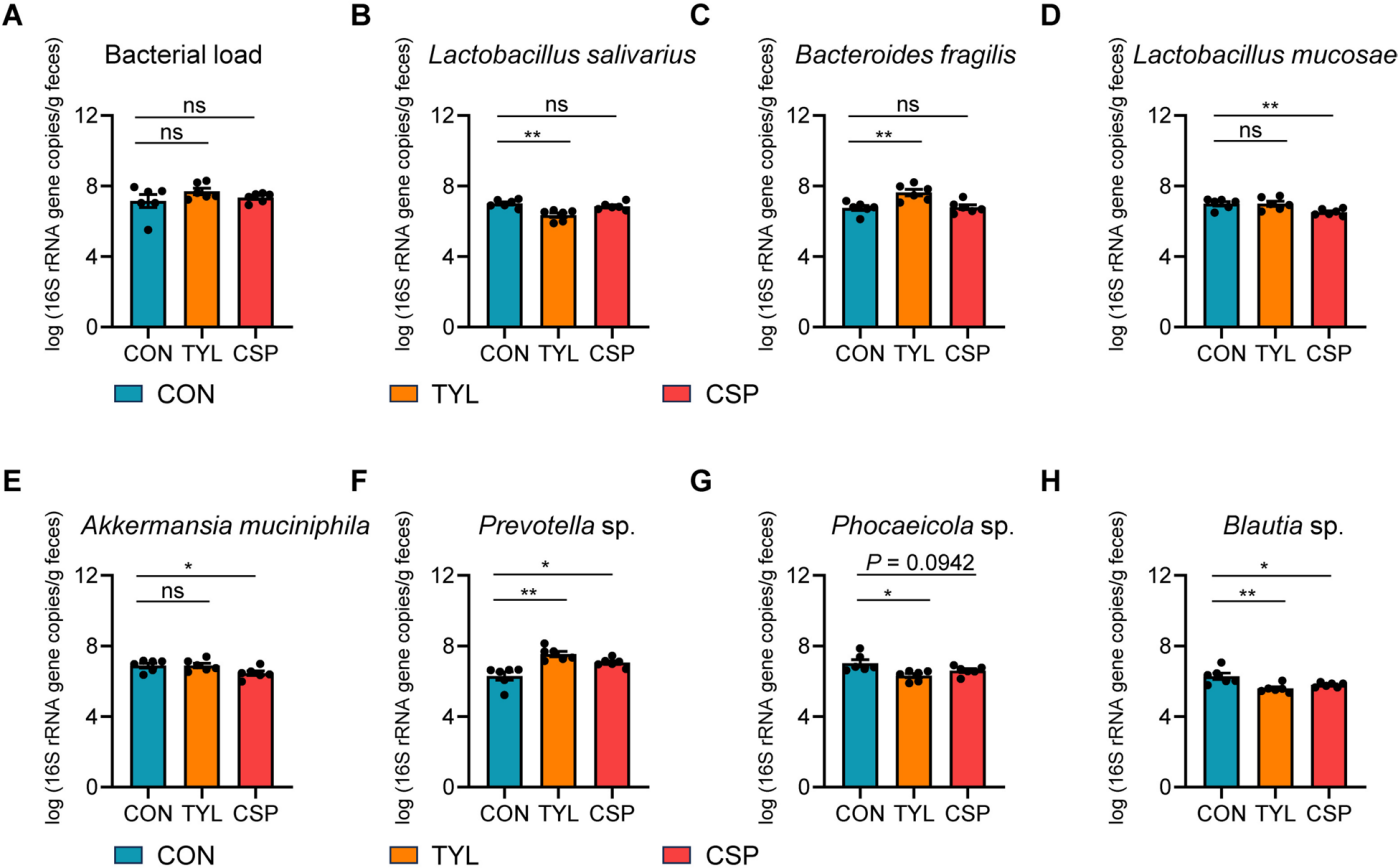
Antibiotic exposure affects intestinal microbiota of weaning piglets. Changes in the fecal microbiota of piglets at d 60 using quantitative real-time PCR. **A** Bacterial load. **B** Relative abundance of *Lactobacillus salivarius*. **C** *Bacteroides fragilis*. **D** *Lactobacillus mucosae*. **E** *Akkermansia muciniphila*. **F** *Prevotella*. **G** *Phocaeicola.* **H** *Blautia*. Data are presented as the means ± SEM, *n* = 6 piglets per group, each dot represents an individual animal. ^ns^*P* > 0.05, ^*^P < 0.05, ^**^P < 0.01, using ANOVA with Tukey’s post hoc test

Although the causal relationship between oral microbiota dysbiosis and systemic diseases has been elusive [19], we sought to explore its connection to the intestinal microbiota, which is highly sensitive to peroral antibiotic administration during weaning [45]. We analyzed changes in the fecal microbiota of piglets at d 60 using quantitative real-time PCR. The results showed no difference in total bacterial load among groups (Fig. 7A, *P* > 0.05). In contrast, TYL treatment significantly reduced the relative abundances of *Lactobacillus salivarius* (Fig. 7B, *P* < 0.01), *Phocaeicola* (Fig. 7G, *P* < 0.05), and *Blautia* compared to the CON group (Fig. 7H, *P* < 0.01), while significantly increasing *Bacteroides fragilis* (Fig. 7C, *P* < 0.05) and *Prevotella* (Fig. 7F, *P* < 0.01). CSP treatment significantly reduced the abundances of *Lactobacillus mucosae* (Fig. 7D, *P* < 0.01), *Akkermansia muciniphila* (Fig. 7E, *P* < 0.05), and *Blautia* (Fig. 7H, *P* < 0.05), while promoting *Prevotella* (Fig. 7F, *P* < 0.05). Notably, *Phocaeicola*, a genus predominant in both the oral cavity and the gut, showed a similar reduction pattern in response to antibiotic exposure during weaning.

**Fig. 8:**
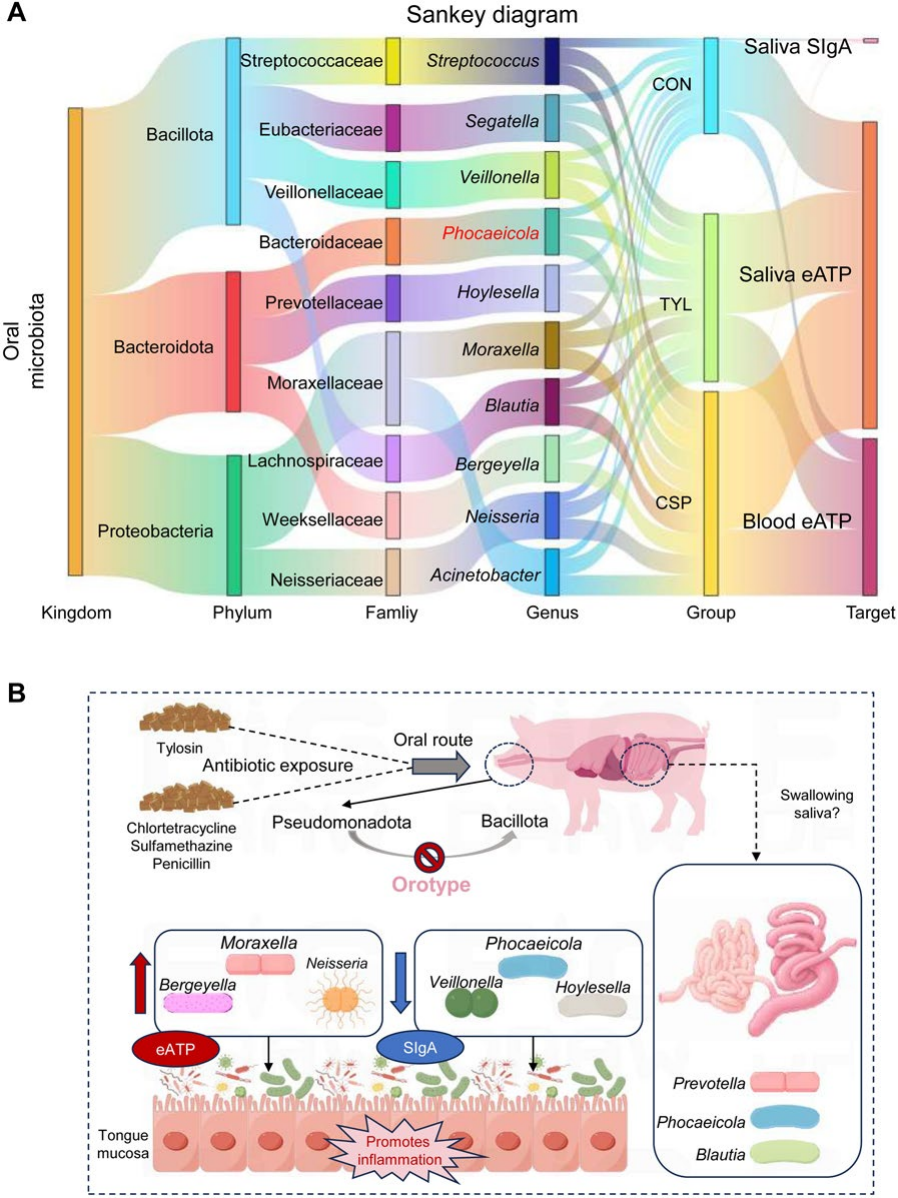
Antibiotic exposure alters oral microbiota composition and correlates with impaired mucosal immunity. **A** Sankey diagram depicting correlations between dominant oral bacterial taxa, experimental groups, and key host physiological parameters. Line widths represent the strength of association, illustrating how microbial shifts induced by TYL and CSP treatments are linked to changes in salivary SIgA and ATP levels. **B** Schematic summary of the proposed mechanism by which antibiotic exposure during weaning disrupts oral microbiota assembly, leading to inflammation, and compromised immune regulation. Created with Figdraw (www.figdraw.com). eATP, extracellular adenosine triphosphate; SIgA, secretory immunoglobulin A

Finally, Sankey diagrams were used to identify key microbial contributors associated with host physiological traits, including salivary SIgA, salivary eATP, and serum eATP levels (Fig. 8A). The plot shows the taxonomic distribution of oral bacteria and their associations with CON, TYL, and CSP groups. Key taxa included Bacillota (*Streptococcus*, *Segatella*, *Veillonella*, *Blautia*), Bacteroidota (*Phocaeicola*, *Hoylesella*, *Bergeyella*), and Pseudomonadota (*Moraxella*, *Neisseria*, *Acinetobacter*).

In the control group, these genera showed the strongest correlations with salivary SIgA, suggesting a role in maintaining the local mucosal barrier. In contrast, under TYL and CSP treatments, these same genera were more strongly correlated with salivary and serum eATP levels. Notably, Moraxellaceae (*Moraxella, Acinetobacter*) were strongly associated with antibiotic exposure. These associations are consistent with shifts in mucosal immune and inflammatory status. Antibiotic treatment also reduced the abundance of *Phocaeicola* (Prevotellaceae) in both oral and gut microbiota. In the CSP group, reduced *Phocaeicola* was accompanied by lower salivary SIgA and higher salivary eATP. Across treatments, *Phocaeicola* abundance was significantly correlated with salivary SIgA, salivary eATP, and serum eATP levels.

Collectively, these findings indicate that antibiotic treatment disrupts the establishment of a ‘normal’ orotype in piglets and alters microbiota composition along the digestive tract, thereby inducing dysbiosis and impairing host physiological homeostasis (Fig. 8B).

## Discussion

The oral microbiota, shaped by host immunophysiology and environmental factors, plays a vital role in maintaining host homeostasis. A shift to a dysbiotic state can provoke local and systemic inflammation, contributing to various diseases through mechanisms that remain incompletely understood. Here, we characterized oral microbiota assembly in piglets, a model chosen for its agricultural relevance, physiological similarities to humans [46], and a shared core resistome [47]. To our knowledge, this is the first study to document the developmental succession of oral microbiota in piglets across the critical weaning stage and assess how early-life antibiotic exposure disrupts this natural microbial assembly.

Consistent with prior work on porcine oral microbiota, we detected dominant *Moraxella* and *Streptococcus* in our samples [48]. Most published studies focus on static community profiles in mature pigs and characterize core microbes of oral mucosal niches, but lack data on microbial dynamics in young animals [17, 49, 50]. Our longitudinal analyses captured a unique developmental trajectory in weaning piglets. Earlier research also reported limited microbiota changes in the tonsils of adult pigs following chlortetracycline treatment [51], whereas the two antibiotic regimens tested here profoundly restructured the oral community and halted its maturation in piglets. This contrast highlights that developing oral microbiota in juveniles is far more sensitive to antibiotics than the stable microbiome of mature swine. Our results therefore advance current understanding by revealing stage-specific microbial succession and the heightened vulnerability of early-life oral microbiota to antimicrobial disturbance.

Intriguingly, we found that the salivary bacterial community began as a "Pseudomonadota" orotype at weaning, driven by Moraxellaceae. In control piglets, this community transitioned and stabilized post-weaning into a "Bacillota” orotype, with Lachnospiraceae and Oscillospiraceae, emerging as keystone, temporally stable families. Critically, this maturation was disrupted by low-dose antibiotic exposure during weaning. Antibiotic treatment prevented the transition to a Bacillota-dominated state, instead enriching for resilient families like Prevotellaceae, Streptococcaceae, Pasteurellaceae, and Moraxellaceae. Antibiotic exposure increased *Moraxella* and *Bergeyella* while reducing *Veillonella*, *Hoylesella*, and *Phocaeicola*, with the latter taxa were also diminished in the gut microbiota. Functionally, these microbial alterations correlated with changes in salivary SIgA, extracellular ATP levels and local immune activation regulation, suggesting that antibiotic-induced dysbiosis disrupts key mechanisms responsible for maintaining host immunophysiological homeostasis.

While antibiotics remain indispensable for treating bacterial infections in farm animals, their overuse as growth promoters drives antimicrobial resistance and poses public health risks [2–4, 6]. Here we evaluated two widely applied swine antibiotic regimens, tylosin and a chlortetracycline-sulfadiazine-penicillin mixture (CSP) [9, 22, 52, 53]. In line with prior reports [22, 54], neither treatment enhanced piglet growth performance, further justifying restrictions on in-feed antibiotics for growth promotion.

More importantly, our study is the first to define a developmental succession of orotype in piglets. A healthy transition occurs from an early "Pseudomonadota" orotype (driven by Moraxellaceae) at weaning to a stable, mature "Bacillota" orotype (driven by Lachnospiraceae) post-weaning. Exposure to either tylosin or CSP arrested this succession, preventing the establishment of the mature Bacillota state. This dysbiosis was functionally linked to disruptions in microbial energy, carbohydrate, and lipid metabolism. Coupled with a concurrent reduction in host systemic metabolic activity, these oral and metabolic impairments likely explain the absence of a growth-promoting effect, underscoring the hidden physiological cost of antibiotic exposure during this critical developmental window.

Antibiotics are hypothesized to promote a pro-inflammatory dysbiosis partly by altering microbial energy harvest [2, 45]. Consistent with this, we observed local inflammation in antibiotic-treated piglets, evidenced by mononuclear cell infiltration in the tongue mucosa and elevated levels of salivary and serum extracellular ATP, a damage-associated molecule that stimulates immune responses [41, 55]. Transcriptomic analysis of oral tissue revealed that this inflammation was underpinned by the upregulation of key immune pathways, including "Cytokine-cytokine receptor interaction" and "Toll-like receptor signaling". Concurrently, a reduction in salivary SIgA was observed, indicating a compromised mucosal barrier and diminished immune control over the microbiota [56]. This loss of regulatory capacity likely permitted the persistence of an immature, Pseudomonadota-dominated orotype post-weaning [57]. This community was enriched in pro-inflammatory genera linked to mucosal barrier impairment, including *Moraxella* [58, 59], *Acinetobacter* [60], and *Neisseria* [61]. The enrichment of *Moraxella* is particularly notable, as it dominates the early-life respiratory microbiota of suckling and newly weaned piglets, representing a hallmark of immature mucosal microbiota [62, 63]. Furthermore, *Moraxella* has been repeatedly associated with mucosal inflammation and disease in piglets [58] and with aging-associated inflammation in adults [59]. In line with these local alterations, our blood biochemistry results also confirmed systemic metabolic disturbances and inflammatory changes in antibiotic-exposed piglets. Furthermore, antibiotic-induced dysbiosis was linked to an upregulation of microbial lipid metabolism pathways. We speculate that this shift may contribute to elevated lipopolysaccharide (LPS) production by Gram-negative pathogens [64]. LPS can in turn trigger abnormal lipid deposition and peroxidation, further exacerbating local and systemic inflammation [65]. Collectively, these findings illustrate that antibiotic exposure converges on shared outcomes, immune activation, barrier impairment, and inflammatory dysbiosis, acting through distinct yet interconnected microbial and metabolic pathways.

A self-perpetuating cycle of dysbiosis can occur when compromised immune function allows opportunistic pathogens to expand, simultaneously depleting beneficial commensals [2, 45]. In our study, chronic antibiotic exposure with tylosin or CSP reduced the relative abundance of key commensal genera in the salivary microbiota, including *Hoylesella*, *Phocaeicola*, and *Veillonella*. The decline of *Veillonella* is particularly relevant. This genus plays a crucial role in metabolic homeostasis by fermenting lactate, produced by bacteria like *Streptococcus,* into weaker acids [66]. Its loss may lead to lactic acid accumulation, creating an acidic microenvironment that impairs the mucosal barrier and facilitates pathogen invasion, consistent with the observed expansion of Pseudomonadota. Similarly, the reduction of *Phocaeicola* may exacerbate dysbiosis. While it also produces short-chain fatty acids alongside oral taxa such as *Veillonella*, *Phocaeicola* primarily contributes to sustained anti-inflammatory status and systemic metabolic health [67, 68]. Notably, we found that antibiotic-induced decreases in *Phocaeicola* occurred in both the oral and gut microbiota. Given that oral administration of *Phocaeicola* can protect against colitis by strengthening the intestinal barrier [68], its depletion may disrupt oral-gut axis homeostasis. Therefore, we postulate that antibiotic-induced shifts in specific oral taxa like *Phocaeicola* not only reflect local dysbiosis but may also serve as biomarkers for broader systemic disruption, highlighting potential targets for probiotic intervention.

This study has several limitations. First, while saliva effectively collects bacteria and metabolites across oral sites [27, 43], it only reflects an overall profile and cannot resolve site-specific microbial differences in the oral cavity. Second, our analysis relied on 16S rRNA gene sequencing, which profiles bacterial composition but not other microbial domains (archaea, fungi, viruses) or functional potential. A more comprehensive understanding will require metagenomic and other multi-omics approaches [15]. Finally, we primarily characterized the oral microbiota, with only a preliminary exploration of its connection to the gut. The existence and mechanisms of a dedicated oral-gut axis in pigs warrant further investigation.

## Conclusions

This study establishes that normal porcine oral microbiota maturation follows a defined successional pattern from an early Pseudomonadota-dominated state to a stable Bacillota orotype post-weaning. We demonstrate that low-dose antibiotic exposure abrogates this program, locking the community in a dysbiotic state marked by a loss of beneficial taxa (e.g., *Veillonella*, *Phocaeicola*) and expansion of opportunistic pathogens. This dysbiosis is mediated by compromised salivary SIgA immunity, altered local metabolism, and the triggering of both local and systemic inflammation. Our findings provide a crucial framework for understanding how early-life antibiotics disrupt oral ecological succession, laying a foundation for biomarkers and strategies to mitigate dysbiosis and promote sustainable swine health.

## Supplementary Information

Additional file 1: Supplementary Table S1. Nucleotide sequences of specific primers used for real-time PCR.

## Data Availability

The 16S rDNA sequencing data in this study was uploaded to GenBank at the National Center for Biotechnology Information with accession number PRJNA1248655. RNA-seq data are available in the SRA under BioProject accession number PRJNA1414931.

## Abbreviations

ALB: Albumin
ALP: Alkaline phosphatase
ALT: Alanine aminotransferase
AMR: Antimicrobial resistance
AST: Aspartate aminotransferase
BUN: Blood urea nitrogen
eATP: Extracellular adenosine triphosphate
GLU: Glucose
HDLC: High-density lipoprotein cholesterol
H&E: Hematoxylin and eosin
IBD: Inflammatory bowel disease
KEGG: Kyoto Encyclopedia of Genes and Genomes
LDH: Lactate dehydrogenase
LDLC: Low-density lipoprotein cholesterol
MAGs: Metagenome-assembled genomes
NEFA: Non-esterified fatty acids
OTU: Operational taxonomic unit
PCoA: Principal coordinate analysis
SIgA: Secretory IgA
TCHO: Total cholesterol
TG: Triglycerides
TP: Total protein
UA: Uric acid
