# Supplementary material for: Antibiotic exposure during weaning disrupts oral microbiota assembly in piglets
Source: J Anim Sci Biotechnol. 2026 Aug 11;17:163. doi: 10.1186/s40104-026-01483-y (PMC13459214; doi:10.1186/s40104-026-01483-y)
Supplement: Supplementary file 1 [file 40104_2026_1483_MOESM1_ESM.docx]

| **Supplementary Table S1**. Primers used in this study | | | |
| --- | --- | --- | --- |
| Target bacteria | Primer | Sequence (5’-3’) | Reference |
| Universal 16S rDNA | F | TCCTACGGGAGGCAGCAGT | ^[1]^ Muyzer et al., 1993 |
|  | R | GGACTACCAGGGTATCTAATCCTGTT | ^[2]^ Klindworth et al., 2013 |
|  | Probe | 6-FAM-CGTATTACCGCGGCTGCTGGCAC-TAMRA | ^[3]^ Nadkarni et al., 2002 |
| *Lactobacillus salivarius* | F | GTCGTAACAAGGTAGCCGTAGGA | ^[4]^ Harrow et al., 2007 |
|  | R | TAAACAAAGTATTCGATAAATGTACAGGTT |  |
| *Bacteroides fragilis* | F | TGAGGCTGTTACGCGATTGA | This study* |
|  | R | GAACTAATGAGGAAAGCCATCGA |  |
| *Lactobacillus mucosae* | F | GAACGCATTGGCCCAA | This study* |
|  | R | TCCATTGTGGCCGATCAGT |  |
| *Akkermansia muciniphila* | F | TACAGCCCCGTTCACTCTCAT | This study* |
|  | R | TTAACGCTGACGGAGAATTGC |  |
| *Prevotella spp.* | F | GTAGGGGTTCTGAGAGGA | ^[5]^ Cannon et al., 2017 |
|  | R | AGCTGCCTTCGCAATCGG |  |
| *Phocaeicola spp.* | F | ACGATGAATACTCGCTGTTTGC | This study* |
|  | R | CATGTTCCTCCGCTTGTGC |  |
| *Blautia spp.* | F | GTGAAGGAAGAAGTATCTCGG | ^[6]^ Kurakawa et al., 2015 |
|  | R | TTGGTAAGGTTCTTCGCGTT |  |

*Primers were designed based on conserved gene regions of target species retrieved from NCBI GenBank (as of March 2024). Their specificity was confirmed by in silico analysis using NCBI Primer-BLAST against the non-redundant nucleotide database (no cross-reactivity with non-target bacteria commonly found in the porcine intestinal microbiota), by melting-curve analysis (single peak), and by agarose gel electrophoresis of PCR products (single band of expected size).

**Reference**

[1]	Muyzer G, de Waal EC, Uitterlinden AG. Profiling of complex microbial populations by denaturing gradient gel electrophoresis analysis of polymerase chain reaction-amplified genes coding for 16S rRNA. Appl Environ Microbiol. 1993;59(3):695-700.

[2]	Klindworth A, Pruesse E, Schweer T, Peplies J, Quast C, Horn M, et al. Evaluation of general 16S ribosomal RNA gene PCR primers for classical and next-generation sequencing-based diversity studies. Nucleic Acids Res. 2013;41(1):e1.

[3]	Nadkarni MA, Martin FE, Jacques NA, Hunter N. Determination of bacterial load by real-time PCR using a broad-range (universal) probe and primers set. Microbiology (Reading). 2002;148(Pt 1):257-266.

[4]	Harrow SA, Ravindran V, Butler RC, Marshall JW, Tannock GW. Real-time quantitative PCR measurement of ileal *Lactobacillus salivarius* populations from broiler chickens to determine the influence of farming practices. Appl Environ Microbiol. 2007;73(22):7123-7127.

[5]	Cannon K, Byrne B, Happe J, Wu K, Ward L, Chesnel L, et al. Enteric microbiome profiles during a randomized Phase 2 clinical trial of surotomycin versus vancomycin for the treatment of Clostridium difficile infection. J Antimicrob Chemother. 2017;72(12):3453-3461.

[6]	Kurakawa T, Ogata K, Matsuda K, Tsuji H, Kubota H, Takada T, et al. Diversity of intestinal *Clostridium coccoides* group in the japanese population, as demonstrated by reverse transcription-quantitative PCR. PLoS One. 2015;10(5):e0126226.
